# Development and user-testing of a brief decision aid for aspirin as a preventive approach alongside colorectal cancer screening

**DOI:** 10.1186/s12911-021-01523-9

**Published:** 2021-05-20

**Authors:** Lenira Semedo, Kate J. Lifford, Adrian Edwards, Kathy Seddon, Kate Brain, Stephanie Smits, Sunil Dolwani

**Affiliations:** 1 Division of Population Medicine, Cardiff University, Cardiff, UK; 2 Department of Gastroenterology, University Hospital Llandough, Penlan Road, Penarth, Cardiff, CF64 2XX UK

**Keywords:** Aspirin, Cancer prevention, Colorectal cancer, Decision-making, Bowel screening

## Abstract

**Background:**

Several epidemiological and cohort studies suggest that regular low-dose aspirin use independently reduces the long-term incidence and risk of colorectal cancer deaths by approximately 20%. However, there are also risks to aspirin use, mainly gastrointestinal bleeding and haemorrhagic stroke. Making informed decisions depends on the ability to understand and weigh up benefits and risks of available options. A decision aid to support people to consider aspirin therapy alongside participation in the NHS bowel cancer screening programme may have an additional impact on colorectal cancer prevention. This study aims to develop and user-test a brief decision aid about aspirin to enable informed decision-making for colorectal screening-eligible members of the public.

**Methods:**

We undertook a qualitative study to develop an aspirin decision aid leaflet to support bowel screening responders in deciding whether to take aspirin to reduce their risk of colorectal cancer. The iterative development process involved two focus groups with public members aged 6074years (n=14) and interviews with clinicians (n=10). Interviews (n=11) were used to evaluate its utility for decision-making. Analysis was conducted using a framework approach.

**Results:**

Overall, participants found the decision aid acceptable and useful to facilitate decision-making. They expressed a need for individualised risk information, more detail about the potential risks of aspirin, and preferred risk information presented in pictograms when offered different options. Implementation pathways were discussed, including the possibility of involving different clinicians in the process such as GPs and/or community pharmacists. A range of potentially effective timepoints for sending out the decision aid were identified**.**

**Conclusion:**

An acceptable and usable decision aid was developed to support decisions about aspirin use to prevent colorectal cancer.

**Supplementary Information:**

The online version contains supplementary material available at 10.1186/s12911-021-01523-9.

## Background

Colorectal cancer is one of the most common cancers in the UK with over 41,000 new cases diagnosed and nearly 16,000 deaths, each year [1]. The biennial faecal occult blood testing prevents colorectal cancer from developing by identifying it at an early stage being associated with a reduction in colorectal cancer deaths of approximately 16% [2]. Currently, the Faecal Immunochemical Test (FIT) is used as standard in the UK Bowel screening programmes. This is due to higher sensitivity of the quantitative FIT test in detecting advanced neoplasia and colorectal cancer, easier handling and assessment and potentially higher uptake [3, 4].

Approaches to reduce colorectal cancer incidence and reduce individual risk have mainly focused on increasing participation in colorectal screening as well as dietary and lifestyle strategies for behaviours such as healthy eating, drinking less alcohol and being more active [3]. A recent approach in primary care focuses on chemoprevention, prescribing low-dose aspirin to lower the risks by delaying and preventing cancer development and mortality [5,7].

The overall benefit from taking low-dose aspirin is similar to screening by colonoscopy for reducing cancer incidence and mortality with aspirin being more effective for proximal colon cancers [5]. Colorectal screening appears most effective for distal colon cancers [6]. Alongside screening, aspirin therapy may potentially have an additional impact on the prevention of colorectal cancer [6, 7]. However, this approach has not yet been tested.

The Australian National Health and Medical Research Council and the United States Preventive Services Task Force (USPTF) recently updated their recommendations based on clinical evidence documenting the effects of daily low-dose (75300mg) aspirin therapy on colorectal cancer incidence and mortality [8, 9]. This evidence derived from pooled analyses of individual patient data in primary and secondary cardiovascular disease and adenoma prevention trials, describing the risks associated with aspirin use, gastrointestinal bleeding and haemorrhagic stroke. These findings indicate a reduction in the incidence of colorectal cancer suggesting a preventive effect of aspirin after 5 to 10years of continued use. The health benefit appears to be largest for adults aged between 50 and 70years [8]. Despite recommendations for population use of low-dose aspirin in prevention of colorectal cancer in some countries [8], this is not currently endorsed in the UK. Further, there is no patient-facing information to facilitate individual decisions. Combining the two-independent risk-reduction strategies, aspirin and colorectal screening, with a decision aid to support informed choice about taking aspirin could optimise behavioural and clinical outcomes in screening-eligible adults [6, 10, 11].

Shared decision-making is a priority for clinicians and policy makers worldwide, and one of the main components of patient-centred care [12]. This is reflected in the development of decision aids, informational tools that support individuals making complex treatment or screening decisions by providing information aligned with individual values and preferences for risk communication [13, 14].

Deciding whether to take aspirin could be supported by shared decision-making between the patient and their clinician, based on individualised risk factors. This would help people weigh up the harms and benefits of taking aspirin as a preventive strategy against colorectal cancer.

Our aim was to develop and user-test a brief decision aid for acceptability and utility, to enable informed decision-making for preventive low-dose aspirin use that could be implemented alongside the UK bowel cancer screening programmes.

## Methods

### Decision aid development and user-testing process

**Fig. 1:**
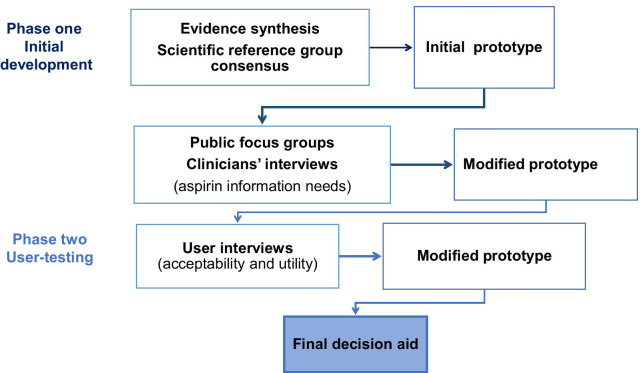
Flow chart showing the iterative process of the decision aid development

The Cardiff University School of Medicine Institutional Review Board granted ethical approval (reference 18/23). The present study was conducted over two phases, development, and user-testing, from September 2018 to August 2019 (Fig.1). In phase one (development), we first identified and summarised the scientific evidence reporting the risks/benefits of taking aspirin to prevent colorectal cancer, derived from existing clinical guidelines/systematic reviews of randomised controlled trials in healthy and clinical populations.

Clinical evidence in summary form was agreed by a scientific reference group of national experts and clinical professionals (n=4). The prototype was developed based on the scientific reference group recommendations and considering the type of information people might need to decide.

Its content was developed according to the International Patient Decision Aids Criteria by presenting clear probabilities of outcomes to help individuals balance options, ensuring decision-making is informed and based on personal preferences [15].

The prototype decision aid was presented in a frequently asked questions format, tailored according to age, three categories (6064; 6569; 7074) within the eligible screening ages and gender. It was then shown to members of the public (focus groups) and clinicians (interviews) for discussion.

The decision aid content and format were further informed by incorporating participants feedback on the draft prototype, information needs, and preferences for risk communication.

Phase two (user-testing) gained user feedback through qualitative interviews with public members. Modifications to the content of the prototype followed an iterative process, informed by participants feedback on acceptability and utility for decision-making in both phases. The content was improved to increase readability and was accredited for clarity of information with a Crystal Mark by the Plain English Campaign [16].

### Patient and public involvement

The present study follows the National Institute for Health and Research UK standards for effective patient and public involvement (PPI) in research [17] (Table 1).

**Table 1:** Standards for effective PPI in research

UK standards for PPI in research	
(a) *Inclusive opportunities*. The Wales Cancer Research Centre has a network of Research Partners (RP) Patients and members of the public who are actively involved in planning and management of research. They offer practical PPI support and advice to researchers. The study involved members of the public for whom the research may prove beneficial (b) *Working together*. PPI input was vital from the very beginning of this research and public-facing materials were developed in collaboration with our PPI partner and co-author (KS) in project management meetings (c) *Support and learning*. PPI contributions were fully recorded and evidenced support and learning in this research, by the RP and by researchers (d) *Communications*. Developing effective methods of communicating and presenting the risks and benefits of taking aspirin to reduce risk of colorectal cancer, in plain language available in formats to suit individual needs was made possible because of the PPI feedback (e) *Impact*. A study summary was made available to participants, the funder (Tenovus Cancer Care) and Bowel Screening Wales. This acknowledged the impact of PPI contributions (f) *Governance*. PPI RP input was integral to this research We also sought public views through discussions with a Citizens Jury [18] who helped explore attitudes towards the role of medicines in maintaining health, using low dose aspirin as a case study, highlighting the importance of providing information to inform decision-making	

### Phase one: brief decision aid development

#### Participants, materials and procedure

##### Members of the public

Inclusion criteria were colorectal screening responders in South Wales, aged 6074years, who could read and speak English. Individuals were recruited through existing community links, Bowel Screening Wales, Healthwise Wales and Tenovus Cancer Care (Appendix).

A covering letter, information sheet and consent form were sent to participants before the focus groups. Participants were allocated to one of two focus groups of mixed age and gender which were audio-recorded.

The focus groups were facilitated by experienced researchers (KL, SS) and started with a brief presentation (LS) explaining the aim of the study, the decision aid concept and the importance of informed decision-making. Subsequently, different paper-based examples of existing decision aids were shown to participants. Finally, participants were given a copy of the aspirin decision aid prototype (large print, A3) and time to process the information before discussing the content, format, usefulness for decision-making, and barriers and facilitators of aspirin use as a preventive strategy for colorectal cancer (see Additional file2-public members focus group topic guide).

##### Clinicians

Clinicians were invited to participate because of their area of expertise and because they may be the first point of contact for some individuals and therefore potentially the ones who may discuss the decision aid with individuals in the future. A covering letter, information sheet and consent form were sent to a purposeful sample of clinicians who were involved in different areas related to the prevention, diagnosis, and management of colorectal cancer, practising in Wales. A copy of the decision aid prototype was sent to clinicians before the interviews to ensure familiarity with the information.

Semi-structured interviews were conducted and audio-recorded either face-to-face in a preferred location (place of work) or, due to practical constraints of time and geographical distance, by telephone. Feedback was sought from clinicians on the usefulness of the decision aid and potential implementation strategies alongside the bowel screening programme (see Additional file 2: Table 2).

### Phase two: user-testing the brief decision aid

#### Participants, materials and procedure

##### Public members

Interview participants were selected purposefully by including a relevant demographic for the screening programme ensuring variation in age, gender and geographic location. Inclusion criteria and recruitment strategies were the same as in phase one. Interviews were conducted according to participants preferences, either face-to-face in a university meeting room or by telephone. Before interviews, participants were sent the refined prototype and two risk pictograms, one with additional figures to represent colorectal cancer and bleeding risks and one without. Interview topic guide (see Additional file 2: user interview topic guide) included information provision, clarity and understanding of information, any sections to be improved, risk pictograms, general format, and views about optimal ways of implementing the decision aid.

#### Data analysis

Data analysis followed the same approach in both phases. A qualitative design was used for data gathering and iterative development of the brief decision aid. Focus group and interview recordings were transcribed verbatim, managed using NVivo [19] qualitative data analysis software (Version 12, 2018) and analysed using a framework approach [20]. Analysis involved (1) familiarisation with the data through comprehensive reading of transcripts, (2) identifying a coding framework by drawing on questions included in the topic guides to capture emerging concepts (LS and KL). Codes related to specific concepts were charted (3) and subsequently summarised, interpreted and any discrepancies resolved by consensus (4).

LS primarily coded the data and a sample (20%) of the transcripts was also coded by KL. Quotes were selected illustrating identified themes. Changes were made to the decision aid based on participant feedback, information needs and preferences for risk communication.

## Results

### Brief decision aid development and user-testing

Focus group participants had a mean age of 64.71years, mostly highly educated, 50% male. Clinicians were of varied backgrounds, 50% male. Interview participants had a mean age of 67.09years, mostly male (60%) and 50% were currently taking aspirin (Table 2).

**Table 2:** Participant demographics and characteristics

Study phase	Phase 1			Phase 2
Participants	Focus group 1 (n=7)	Focus group 2 (n=7)	Clinician interviews (n=10)	Public interviews (n=11)
*Age* Mean (range) years	64.71 (6169)	66.57 (6372)	n/a	67.09 (6174)
*Gender*				
Male	3	4	5	6
Female	4	3	5	5
*Highest level of education*			n/a	
Bachelors degree/masters/PhD	6	6		3
Further education but not degree	1	1		3
Finished school at or before age fifteen				2
Completed A-levels or equivalent				1
CSEs, O-levels or equivalent				1
Missing				1
*Employment status*			n/a	
Employed full-time	1	2		
Employed part-time	1			2
Retired	5	5		8
Missing				1
*Occupation*	n/a	n/a		n/a
Community pharmacist			2	
Consultant gastroenterologist			3	
General practitioner			2	
Specialist screening practitioner			3	
*University Health Board*	n/a	n/a		n/a
Aneurin Bevan			1	
Cardiff & Vale			7	
Cwm Taff			1	
Hywel Da			1	
*Home living arrangements*			n/a	
Own outright	7	7		9
Rent from local authority/housing association				1
Missing				1
*Aspirin use*			n/a	
Yes		1		5
No	7	6		5
Missing				1
*Area*				
*South Wales*	7	7	10	6
*North Wales*				5

*n/a* not applicable

### Phase one: development

#### Public members feedback on the decision aid prototype

Participants understood the content of the prototype in terms of the risks (bleeding events) and benefits of taking aspirin (reduction in colorectal cancer cases). Some expressed concerns over the severity of risks and perceived reduced benefits, whilst others did not. Participants perceived information gaps in the prototype that needed addressing to increase the utility of the decision aid. These included the consequences of bleeding events, further information on low-dose aspirin (e.g. dose, side effects, and optimum age to start aspirin) and sources of available clinical evidence used to develop the prototype and mortality information (see Additional file 2: Table 1).

In terms of preferences for risk communication, there was consensus that risk information should be changed to pictograms to decrease the amount of text in the prototype. Participants appreciated that the prototype was personalised for age and gender but stated that individual tailoring for specific factors such as family history and medical history would be helpful.

Participants also mentioned wanting to discuss individual risk profiles with a clinician.

I think that without that [ tailored risk], I would want to be discussing it with my doctor you can only answer it on a statistical average basis because everyones colorectal cancer [risk] will be different. (Male, FG1, Participant 2).

Participants suggested simplifying the language and developing supplementary information (e.g. website link) for people who desired to know more.

Views differed when considering implementation pathways alongside the bowel screening programme. Whilst some believed that people could benefit more if the decision aid was introduced before the eligible screening age, e.g. through a public health campaign, others thought that sending the aspirin information alongside the screening invitation could encourage people to consider both in tandem. Individuals also stated that they would want to discuss with other healthcare professionals (e.g. community pharmacists) to relieve pressures on GPs time.

When exploring potential decisions to take aspirin, given the information provided, most individuals said that they would not take aspirin for now but would discuss their options with their GP in the future. Some expressed confidence in their doctors to support them making future decisions.

Generally, participants thought that the decision aid was useful to inform decision-making.

I think it covers the main points for most people it does help them to make their own decision. I dont like taking tablets, thats absolutely fine, but it makes you think of the points youve got to consider. (Female, FG1, Participant 3).

Most participants were satisfied with the short leaflet format, although others thought that a booklet format would help to present the information. Some preferred a paper-based prototype whilst others would rather access information online.

#### Clinicians feedback on the prototype

Clinicians commented on specific information they felt was useful to include to improve decision quality, building on the information requested by public members (see Additional file 2: Table 2). This included further information about aspirin stating the consequences of bleeding risks and using simpler language to explain information. Clinicians felt it was important to clarify the purpose of information to potential users. Some thought the risk information was well presented, and others felt that pictograms would increase the level of engagement with the information.

A few clinicians thought that risk stratification was a complex subject to address due to the impact of other factors on ones level of risk.

it is a very much averageI dont think you can give a very specific risk to every single person that youll see, because youve got factors such as age, diet, lifestyle, family history and also things like past medical history, other treatments theyre taking (Consultant gastroenterologist 1)

They believed that the prototype included useful information for people to decide whether to take aspirin and conveyed the need to develop such information for peoples benefit.

They thought that the format/length of the prototype was good, but it would be useful to break it down into sections to facilitate its use.

Discussions about implementation pathways concerned the best timing to send the decision aid, shared decision-making, and potential barriers to implementation. Clinicians thought that the information should be communicated through primary care, others said it could be sent with the initial bowel screening invitation or after a positive screening test result. They also agreed that the GP would represent a safety net to discuss options but that it would be important for different clinicians to be involved, by making every contact count[21]. Most clinicians felt confident to discuss the information though some felt that they would need more information about aspirin to engage in shared decision-making. Potential barriers to implementation included addressing time constraints and resourcing for clinicians.

### Phase two: user-testing

#### Users feedback on the modified prototype

The modified prototype was received positively by potential users. Overall, participants thought that it was concise, clear and understandable with the right level of language.

Minor improvements to the content were suggested to supplement the information in the prototype such as including information for people already using aspirin and more information on bleeding events (see Additional file 2: Table 3). Whilst participants thought that risk information was conveyed well, they preferred the pictogram with no images and suggested presenting the risks of colorectal cancer and bleeding separately.

Generally, participants were pleased with the modified prototype. Only one participant was unsure about how the decision aid would be used.

I think I least like this sort of confusion about whether you could just take it or go and see the GP (Female, Participant 2)

The strengths of the modified prototype were discussed and included personalisation to age groups.

I like how its presented for my age group its more directive to me (Male, Participant 11)

Participants welcomed the information regarding the importance of considering both screening and aspirin as strategies for colorectal cancer risk-reduction and thought that both aspects would likely influence future screening uptake.

A few participants who were taking aspirin for other reasons stated that the information reassured them to continue taking aspirin.

so, what I quite liked about it was that this has sort of reassured me to carry on taking aspirin so I personally found it useful. (Male, Participant 6)

Others mentioned that they would further discuss it with their clinician due to health problems or because they disliked taking medication. Some said that they would be inclined to take it if it benefitted them.

Participants were pleased with the modified prototype format in terms of layout, length, order, graphics, font size, colours. They thought it followed a logical structure by explaining and presenting options that would support potential decisions.

As in phase one, there were different views regarding the best time point to implement the decision aid.

#### The user-tested final decision aid

**Fig. 2:**
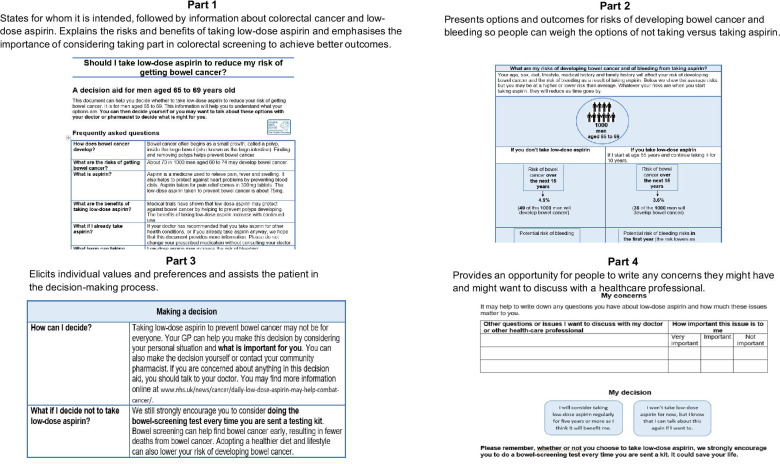
Different sections of the final decision aid informed by the latest clinical evidence

The brief decision aid (Additional file 1-available on request) is structured in four main parts over four A4 pages (Fig.2).

## Discussion

In the absence of information for the use of low-dose therapeutic aspirin use to support decision-making, we developed and tested a brief decision aid presenting the risks and benefits of aspirin as a preventive strategy for colorectal cancer, which could be used and implemented in current population and healthcare settings. The decision aid was developed iteratively, by including the best available clinical evidence and its content and format were modified based on participants information needs and preferences for risk communication. Implementation strategies were discussed, including the best timing to send the decision aid and the need to involve different clinicians in the process. However, there were different views regarding when to implement the decision aid.

Strengths of the decision aid relate to the rigorous development process which followed International Patient Decision Aid guidelines [15, 23]. Likewise, public and patient input adhered to good practice [17]. The decision aid was edited to improve user understanding and accredited with a Crystal Mark by the Plain English Campaign [16]. The decision aid content was tailored based on age and gender, however, participants wanted individualised risk information. When considering decisions about medication use, the benefits may not clearly outweigh the risks for all patients, therefore individuals were encouraged in the decision aid to directly discuss options with their GP or community pharmacist.

We acknowledge potential limitations in this development study. The sample included individuals who could read and speak English and educated to degree level, potentially not entirely representative of people receiving this information in the future. Wider field-testing of the decision aid should therefore involve a more diverse group of users and potentially be adapted for use with underserved groups (e.g. low literacy) to reduce inequalities in healthcare communication. Further refinement of the decision aid may also consider specific groups who may benefit from aspirin therapy.

The decision aid was designed to include balanced information whilst avoiding cognitive burden [25]. Participants requests for key information were mostly fulfilled and, where appropriate, signposting to the product information (e.g. side effects) applied. Additional multilevel material on a website or paper may be developed to explain specific themes.

Existing studies on aspirin use report mixed findings that merit further investigation [26,30]. The USPTF guidelines suggest routine prescribing of aspirin in patients presenting with advanced colorectal polyps unless contraindicated [9]. Despite this, studies still report low aspirin use in these populations [26]. Other evidence suggests that aspirin may impact on FIT performance. Studies have reported either a potential higher risk of false positive FIT results, no changes in FIT diagnostic accuracy in patients presenting with gastrointestinal symptoms or an increase in FIT sensitivity by aspirin use [27,30]. There is also uncertainty about the optimum dose for low-dose aspirin use in the prevention of colorectal cancer. Available evidence suggests that an effective dose of aspirin would depend on factors such as body weight [31].

To our knowledge, this is the first study to report the development and user-testing of a decision aid for aspirin to prevent colorectal cancer for a bowel screening population. It is vital to consider implementation strategies including alongside a screening programme. The decision aid could be used without the presence of a clinician and/or as a component of shared decision-making in a primary care setting by ensuring tailored information is communicated effectively and potentially in combination with individualised risk calculators [32,35]. If the decision aid is sent with the initial screening invitation or following a positive test result, discussions could involve the GP and/or community pharmacist or specialist screening practitioner/colonoscopist, respectively. Resources such as education, training needs and costs would be required to successfully embed the decision aid within the bowel screening programme.

Implementation strategies for the decision aid warrant further investigation which could be done by field testing the decision aid. Examination of how the decision aid might influence behavioural intentions (aspirin use and screening) and its impact on screening uptake also need to be explored. A recent Australian study investigated specific risk presentation formats and intentions to take aspirin to prevent colorectal cancer but did not examine understanding or attitudes to aspirin use or screening intentions [36].

In the future, the evaluation of a combined risk-reduction strategy of aspirin, screening, diet and lifestyle may directly benefit subsequent screening invitees in terms of improved behavioural and clinical outcomes.

## Conclusions

An acceptable and usable decision aid was developed to support decisions about aspirin use to prevent colorectal cancer. Field testing of the decision aid would assess the feasibility of its use alongside the bowel screening programme.

### Supplementary Information

**Additional file 1.** Pre-field-testing version Cardiff Aspirin Decision Aid for the prevention of colorectal cancer.

**Additional file 2.** Development and user-testing of a brief decision aid for aspirin as a preventive approach alongside colorectal cancer screening.

## Data Availability

The datasets used and/or analysed during the current study are available from the corresponding author on reasonable request.

## Appendix

*Bowel Screening Wales* is responsible for the NHS bowel screening programme in Wales which is currently offered to individuals aged 6074years old. The screening engagement team (SET) work across Wales to improve knowledge and raise awareness of screening within local communities. This involves delivering community training, providing resources, attending networking events and running local initiatives with third-sector organisations across Wales. The team has links to participant networks to improve the development of public information, service user experience and screening uptake across all the screening programmes in Wales.

The *Tenovus Cancer Care research* network is a 500 strong list of individuals who have been diagnosed with or indirectly affected by cancer (patients, carers) including people who have an interest in research with no background of cancer. The research network are sent information about studies every four months. Potential participants will register interest via the telephone number or email address provided in the newsletter. Information about studies being carried out are sent through newsletters.

*Healthwise Wales* (HHW) represents a project funded by Health and Care Research in Wales aimed at improving the health and well-being of the population in Wales. The project team includes Cardiff and Swansea universities (SAIL and UKSerRP), health and care research Wales and patient and public involvement to involve public members in consultation and collaboration with project management. Healthwise Wales has a register of over 40,000 who are part of a cohort of longitudinal data on health, lifestyle and health services linked to routinely available healthcare data in Wales. People are sent information approximately every 6months about opportunities to engage in research across five main themes (1) impact of social inequalities on health and well-being (2) environment, neighbourhood and health, (3) maintenance of health and well-being in the working age population (4) wellbeing in later life and (5) innovation in health and social care services. Patient and public involvement.

## Abbreviations

FIT: Faecal immunochemical test
PPI: Patient and public involvement
RP: Research partner
USPTF: United States Preventive Task Force
