# Supplementary material for: Development and user-testing of a brief decision aid for aspirin as a preventive approach alongside colorectal cancer screening
Source: BMC Med Inform Decis Mak. 2021 May 20;21:165. doi: 10.1186/s12911-021-01523-9 (PMC8139147; doi:10.1186/s12911-021-01523-9)
Supplement: Supplementary file 1 — Additional file 1. Pre-field-testing version Cardiff Aspirin Decision Aid for the prevention of colorectal cancer. [file 12911_2021_1523_MOESM1_ESM.pdf]

Should I take low-dose aspirin to reduce my risk of getting bowel cancer?

A decision aid for men aged 65 to 69 years old

This document can help you decide whether to take low-dose aspirin to reduce your risk of getting bowel cancer. It is for men aged 65 to 69. This information will help you to understand what your options are. **You can then decide yourself or you may want to talk about these options with your doctor or pharmacist to decide what is right for you.**

Frequently asked questions

**How does bowel cancer develop?**	Bowel cancer often begins as a small growth, called a polyp, inside the large bowel (also known as the large intestine). Finding and removing polyps helps prevent bowel cancer.
**What are the risks of getting bowel cancer?**	About 70 in 1000 men aged 60 to 74 may develop bowel cancer.<br><br>About 50 in 1000 women aged 60 to 74 may develop bowel cancer.
**What is aspirin?**	Aspirin is a medicine used to relieve pain, fever and swelling. It also helps to protect against heart problems by preventing blood clots. Aspirin taken for pain relief comes in 300mg tablets. The low-dose aspirin taken to prevent bowel cancer is 75mg.
**What are the benefits of taking low-dose aspirin?**	Medical trials have shown that low-dose aspirin may protect against bowel cancer by helping to prevent polyps developing. The benefits of taking low-dose aspirin increase with continued use.
**What if I already take aspirin?**	If your doctor has recommended that you take aspirin for other health conditions, or if you already take aspirin anyway, we hope that this document provides more information. Please do not change your prescribed medication without consulting your doctor.
**What harm can taking low-dose aspirin do?**	Low-dose aspirin may increase the risk of bleeding:<br>• from the stomach, leading to blood in vomit or black or tarry stools (poo); and,<br>• **rarely** bleeding into the brain, usually in the form of a stroke.<br><br>The risk of bleeding reduces as time goes by. Bleeding can also happen in people not taking low-dose aspirin (see the diagram on the next page).<br><br>There are also other minor side effects. These are explained in the leaflet that comes with the medication.

Please turn over

**If I take low-dose aspirin, do I still need to do bowel-screening tests every two years?**	Yes. Whether you choose to take low-dose aspirin or not, we still strongly encourage you to consider **doing the bowel-screening test every time you get a testing kit**. Evidence suggests that if you do bowel-screening tests and take low-dose aspirin, there is a lower risk of you developing and dying from bowel cancer.
**When and how would I need to take low-dose aspirin, if I decide to?**	You would take a low-dose aspirin tablet (standard, dispersible or coated) every day after food. It may be prescribed by your doctor or you can buy it at pharmacies. Your doctor or pharmacist will discuss what dose is right for you (as this may depend on your weight).
**How long would I need to take low-dose aspirin for to benefit?**	The benefits of taking low-dose aspirin increase over time and are greater in people aged 50 to 70. You would need to take low-dose aspirin for **five or more years to reduce your risk** of getting bowel cancer. The benefits of taking low-dose aspirin may last for a further five to 10 years after you stop taking it.<br><br>Evidence of the benefit of taking low-dose aspirin after the age of 70 is less clear.

**What are my risks of developing bowel cancer and of bleeding from taking aspirin?**	
Your age, sex, diet, lifestyle, medical history and family history will affect your risk of developing bowel cancer and the risk of bleeding as a result of taking aspirin. Below we show the average risks, but you may be at a higher or lower risk than average. Whatever your risks are when you start taking aspirin, they will reduce as time goes by.	
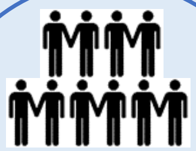<br>**1000 men aged 65 to 69**	
**If you don't take low-dose aspirin**	**If you take low-dose aspirin**<br>If I start at age 65 years and continue taking it for 10 years.

Risk of bowel cancer **over the next 15 years**<br>↓<br>**4.9%**<br>(**49** of the **1000** men will develop bowel cancer)	Risk of bowel cancer **over the next 15 years**<br>↓<br>**3.6%**<br>(**36** of the **1000** men will develop bowel cancer)
Potential risk of bleeding<br>**Less than 0.5%**<br>(Fewer than **five** of the **1000** men will bleed from the stomach)<br>**Less than 0.2%**<br>(Fewer than **two** of the **1000** men will bleed into the brain)	Potential risk of bleeding risks **in the first year** (the risk lowers as time goes by)<br>**0.7%**<br>(**Seven** of the **1000** men will bleed from the stomach)<br>**Less than 0.3%**<br>(Fewer than **three** of the **1000** men will bleed into the brain)

Despite a slightly higher risk of bleeding, the risks of dying from bowel cancer during your lifetime are reduced by 20% in those taking low-dose aspirin when compared with those not taking low-dose aspirin.

**Making a decision**	
**How can I decide?**	Taking low-dose aspirin to prevent bowel cancer may not be for everyone. Your GP can help you make this decision by considering your personal situation and **what is important for you**. You can also make the decision yourself or contact your community pharmacist. If you are concerned about anything in this decision aid, you should talk to your doctor. You may find more information online at www.nhs.uk/news/cancer/daily-low-dose-aspirin-may-help-combat-cancer/.
**What if I decide not to take low-dose aspirin?**	We still strongly encourage you to consider **doing the bowel-screening test every time you are sent a testing kit**. Bowel screening can help find bowel cancer early, resulting in fewer deaths from bowel cancer. Adopting a healthier diet and lifestyle can also lower your risk of developing bowel cancer.

Please turn over

My concerns

It may help to write down any questions you have about low-dose aspirin and how much these issues matter to you.

**Other questions or issues I want to discuss with my doctor or other health-care professional**	**How important this issue is to me**		
	Very important	Important	Not important

My decision

I will consider taking low-dose aspirin regularly for five years or more as I think it will benefit me.

I won't take low-dose aspirin for now, but I know that I can talk about this again if I want to.

Please remember, whether or not you choose to take low-dose aspirin, we strongly encourage you to do a bowel-screening test every time you are sent a kit. It could save your life.

This decision aid was developed by a team of specialists at Cardiff University, working with Bowel Screening Wales. The scientific evidence it is based on is from systematic reviews of controlled medical trials and international evidence-based recommendations. The content was confirmed by a scientific reference group to make sure it provides the best current evidence.

This decision aid was funded by Tenovus Cancer Care.
