# Supplementary material for: Development and user-testing of a brief decision aid for aspirin as a preventive approach alongside colorectal cancer screening
Source: BMC Med Inform Decis Mak. 2021 May 20;21:165. doi: 10.1186/s12911-021-01523-9 (PMC8139147; doi:10.1186/s12911-021-01523-9)
Supplement: Supplementary file 2 — Additional file 2. Development and user-testing of a brief decision aid for aspirin as a preventive approach alongside colorectal cancer screening. [file 12911_2021_1523_MOESM2_ESM.docx]

**Page n.**

1. Public members interview topic guide ………………………………………………………………………………………. 1

2. Clinicians’ interview topic guide ………………………………………………………………………………………………….2

3. User interview topic guide …………………………………………………………………………………………………………..2

4. Table 1. Information gaps in the prototype highlighted by public members…………………………………3

5. Table 2. Feedback from clinicians about the prototype and its potential use……………………………….4

6. Table 3. Users’ feedback on the modified prototype and its potential use……………………………………5

**1. Public members interview topic guide**

1. *Barriers and facilitators to aspirin as a preventive strategy (e.g. Risks and benefits; what information would be useful to include)*
2. *Language (e.g. Understanding)*
3. *Views on the possible usefulness of a brief decision aid (e.g. How/when information would be best received).*
4. *Ways of accessing information (e.g. Different formats)*
5. *Making a decision (e.g. What would be important to make a decision*)
6. *Barriers and facilitators to aspirin as a preventive strategy (e.g. information gaps)*
7. *Information presentation style and format (e.g. different formats)*
8. *Implementation (e.g. usefulness; pathways to implementation; potential barriers; shared decision-making).*

**2. Clinicians’ interview topic guide**

**3. User interview topic guide**

1. *Understanding of decision aid (e.g. purpose; different sections)*
2. *Content and layout (e.g. language; visuals; length of information)*
3. *Improvements/usefulness (e.g. most liked/disliked; changes)*
4. *Best timing to receive the decision aid*
5. *Making a decision (e.g. potential decision based on information and what led to decision)*

**4.** Table 1. Information gaps and potential use of the prototype highlighted by public members

| **Theme** | **Illustrative quotes** |
| --- | --- |
| **Time to benefit** | *‘... is there an* ***optimum age*** *[to start aspirin] first…and secondly* ***is there an age at which it’s too late to be effective****?’ (Male, FG1, Participant 3)* |
| **Aspirin dose and medicinal forms** | .*’.it just says an aspirin tablet,* ***it doesn’t tell you what dose****, so I’d like to know that... whether it was* ***coated*** *to protect my stomach…’* (Female, FG1, Participant 4)  *‘…I haven’t seen the word* ***dispersible*** *here,* ***I take daily dispersible*** *[aspirin]...’ (Male, FG2, Participant 5)* |
| **Severity of bleeding risks** | ‘…***how serious is bleeding from the stomach, how serious is bleeding into the brain****, I’d want to know these sort of things.’* (Male, FG1, Participant 1). |
| **Tailored risk** | *‘…****you don’t know what your base point*** *is when you look at the risk of taking aspirin …****it’s harder to make a decision’*** (Female, FG2, Participant 6) |
| **Clinical evidence** | ***‘I want to know more about what studies have taken place,*** *these types of things are* ***useful supporting information*** (Male, FG2, Participant 5) |
| **Colorectal cancer mortality** | ***‘…****In the great detail of* ***mortality risks****, where does colorectal cancer fit in, I mean that’s both from a personal point of view and from an effective use of health service resources…’* *(Male, FG2, Participant 5)* |
| **Presentation of risk** | *‘…I do understand the data, but I would rather see, the* ***pictorial*** ***method of it****...’* (Male, FG2, participant 1) |
| **Language** | *‘…It’s a bit* ***vague*** *whether you carry on taking it [aspirin] forever… what happens do you stop,* ***it doesn’t make it very clear*** *and I am confused about what you do…’* (Female, FG1, participant 4) |
| **Format** | *‘…I think coming out from these focus groups I often find* ***frequently asked questions quite a useful thing*** *to have, to* ***really pick up the ones*** *[questions] that were worrying people the most.’* (Male, FG2, participant 6) |
| **Implementation** | *‘I think common sense would say to* ***put it in the colorectal screening kit if*** *it was to go ahead*.’ (Female, FG1, participant 4) |
| **Shared decision-making** | *‘it’s important* ***that other healthcare professionals*** *are briefed on this as well****, so we take the load off GPs.****’* (Male, FG2, participant 3)  ‘… *I’m not saying we should go back to being paternalistic and telling people what to do but* ***I sometimes think we think we can make more informed decisions than perhaps we can****… I don’t see my GP very often, I just say, bottom line what would you do in my position* (Male, FG1, Participant 1) |

**5.** Table 2. Feedback from clinicians about the prototype and its potential use

| **Theme** | **Illustrative quotes** |
| --- | --- |
| **Aspirin dose** | *‘…****That’s actually talking about a very different dosage of aspirin*** *[used for pain relief] … I think some* ***specificity there is probably important, in terms of dosage****. If we called it* ***75mg aspirin there’s very little way patients could get confused in the future****.’* (Community Pharmacist 1) |
| **Severity of bleeding risks** | *‘… I’d want to know a little bit more about* ***what sort of bleeding. Where does the bleeding happen?****’* (Specialist Screening Practitioner 3) |
| **Mortality** | *‘I think it's important that once you mention there’s one or two possible bleeds into brain, what could be* ***fatal’*** *(*Consultant Gastroenterologist 3*)*  *‘…****it’s a perennial debate*** *about any information, that you give out to any patient,* ***do you include?****[mortality]..because* ***everything that we do has got a risk of death*** *and becomes part of it…if* ***it is a common consequence something clearly needs to be mentioned****…’ (*Consultant Gastroenterologist 1*)* |
| **Presentation of risk** | *‘I think they [public members] might see that [decision aid] and it* ***might be a little bit wordy****. So maybe having a* ***visual to say right that's me out of that 1,000 people potentially I think that might just help them a little bit as well’*** (Specialist Screening Practitioner 2) |
| **Language and format** | *‘… I think there’s probably just perhaps a bit of* ***finessing of some of the wording*** *and the* ***presentation****, just to make it a little easier...’* (Consultant Gastroenterologist 1)  *‘… you could have* ***visuals****… and you could bring out the figures for the risks and benefits, so they jump out a little bit more… again... I’d ask a patient group whether they find that more engaging. But the actual raw data here, I think stands out very well.’* (Consultant gastroenterologist 1) |
| **Implementation** | *‘…they could just* ***send the leaflet when they’re sending the [screening] pack for patients****… the other is to* ***promote through primary care’*** (General Practitioner 1) |
| **Shared decision-making** | *“…****you do need to set up systems where people can gather to discuss it more****… you absolutely have to* ***include the GP as a source of consultation****.’* (Community Pharmacist 1) |
| **Usefulness** | *‘…if we want patients to benefit from taking aspirin, they do need a decision aid ...****I do think it's a useful thing to have****…’ (Consultant gastroenterologist 2)* |

**6.** Table 3. Users’ feedback on the modified prototype and its potential use

| **Themes** | **Illustrative quotes** |
| --- | --- |
| **Aspirin availability** | ***‘…****whether aspirin would be* ***available on prescription****,* ***or whether people would have to pay for it****… because some people can’t just afford to buy things’* (Participant 7, female) |
| **Aspirin use** | *‘…* ***so, people who already use aspirin****…* ***they might want to know a little bit more****… either taking the same dosage or reducing it…’* (Participant 1, male) |
| **Bleeding from the stomach** | *‘…* ***how will I know*** *that* ***my stomach is bleeding****, if you see what I mean?’ (Participant 2, male)* |
| **Pictogram** | *‘…harms to be separated in lines [ risks of colorectal cancer and bleeding] …****the pictures are taking my eyes from the real issue****…’ (Participant 1, Male)* |
| **Overall format** | *‘…I think it is very* ***short*** *[length of decision aid] and concise’ (Participant 6, male)*  *“I think it’s very good and clear, and it sets out to people what the risks are, of not taking it, or of taking it. So, it gives them all the information in a very nice* ***understandable format*** *they need in order to make the decision…I think it is a very good tool’ (Participant 2, female)* |
| **Implementation** | *‘…****It’s best to send it before*** *[the screening age].* ***I think going to the GP is becoming more and more difficult****.* ***So, you’ve got to bring that in... a leaflet on health and wellbeing…****you could have a slot there if you are concerned about colorectal cancer’. Whether you could* ***incorporate it with other health professionals*** *or something … the pharmacist…* *could serve me, “Why are you taking aspirin?”, “Did you know there are benefits of”. (Participant 1, male)* |
